# Intranasal epinephrine in dogs: Pharmacokinetic and heart rate effects

**DOI:** 10.1002/prp2.587

**Published:** 2020-04-17

**Authors:** Kenneth L. Dretchen, Zack Mesa, Matthew Robben, Desmond Slade, Scott Hill, Claire Croutch, Kyle Kappeler, Michael Mesa

**Affiliations:** ^1^ Mesa Science Associates, Inc. Frederick MD USA; ^2^ Robben Consulting, LLC Rochester NY USA; ^3^ MRIGlobal Kansas City MO USA; ^4^ Citoxlab Stilwell KS USA

**Keywords:** administration, anaphylaxis, epinephrine, intranasal, nasal absorption, pharmacokinetics

## Abstract

Epinephrine is the standard of care for the treatment of severe allergy and anaphylaxis. Epinephrine is most often administered through the intramuscular (IM) route via autoinjector. The current study aimed to evaluate an alternative method of epinephrine treatment through intranasal (IN) delivery in dogs. The pharmacokinetic (PK) parameters of maximum plasma concentration (C_max_), time to reach maximum plasma concentration (T_max_), and area under the plasma concentration‐time curve from 0 to 90 minutes (AUC_0–90_) were observed after IN epinephrine (2, 3, 4, 5, 10, and 20 mg) and IM epinephrine via autoinjector (0.15 and 0.3 mg) for 90 minutes. Heart rate effects were measured after IN (2 and 5 mg) and IM (0.15 and 0.3 mg) epinephrine administration. IN epinephrine (5 mg) demonstrated significantly greater plasma epinephrine concentration at 1 minute as compared with IM epinephrine (0.3 mg) (1.68 ± 0.65 ng/mL vs 0.21 ± 0.08 ng/mL, *P* = .03). There were no significant differences in C_max_, T_max_, and AUC_0–90_ between 2‐mg IN and 0.15‐mg IM epinephrine or between 5‐mg IN and 0.3‐mg IM epinephrine. IN epinephrine reduced heart rate increases, as compared to IM epinephrine. IN and IM epinephrine were both well‐tolerated. Overall, IN epinephrine demonstrated advantages over IM epinephrine, including the rapid increase in plasma epinephrine and lack of increased heart rate over time.

**Abbreviations**
AUC: area under the plasma concentration‐time curve
bpm: beats per minute
C_max_: maximum plasma concentration
ECG: electrocardiogram
IM: intramuscular
IN: intranasal
LC‐MS/MS: liquid chromatography tandem‐mass spectrometry
PK: pharmacokinetic
QC: quality control
SMBS: sodium metabisulfite
T_max_: time to reach maximum plasma concentration
USDA: United States Department of Agriculture

## 1 INTRODUCTION

Anaphylaxis is a serious generalized allergic or hypersensitivity reaction that is rapid in onset and potentially fatal, affecting nearly 5% of the population in the United States.^1^, ^2^ The manifestations of anaphylaxis include an acute onset with affectation of the skin or mucosa in the form of hives, flushing, or swelling; compromised breathing with possible dyspnea and wheezing; and arterial hypotension with the potential for syncope.^3^ Immediate epinephrine treatment in anaphylaxis prevents life‐threatening effects and leads to decreased airway resistance, bronchodilation, reversal of hypotension, and protective chronotropic and ionotropic cardiac effects.^1^

Epinephrine is most commonly administered via intramuscular (IM) injection via an autoinjector.^4^, ^5^, ^6^ Autoinjectors are effective in reducing anaphylactic symptoms; however, they are associated with several patient concerns and are often underused.^7^, ^8^, ^9^ Patients and caregivers may lack confidence with autoinjectors, which can contribute to anxiety and impede autoinjector use.^10^, ^11^, ^12^ In addition, there is potential for injuries associated with accidental injections and lacerations at the injection site, as well as the administration of expired autoinjector epinephrine.^5^, ^7^, ^9^, ^13^, ^14^ Furthermore, there have been product recalls due to issues associated with autoinjector malfunction and inadequate drug delivery.^15^ Lastly, cost effectiveness and autoinjector availability are potential barriers for certain patients with severe allergy.^12^, ^16^ These patient concerns may lead to lack of or delayed epinephrine treatment, which decreases the rate of survival in the event of anaphylaxis.^17^

To overcome these drawbacks, alternative routes of administration and devices are needed for epinephrine delivery. The nasal cavity is an ideal site for drug delivery, particularly for medications requiring a rapid onset of action, because of the degree of vascularization and tissue permeability.^18^ Intranasal (IN) administration has been explored for a wide variety of drugs, including naloxone, nalmefene, fentanyl, hydromorphone, midazolam, glucagon, and haloperidol.^19^, ^20^, ^21^, ^22^, ^23^, ^24^, ^25^, ^26^ For example, IN glucagon for hypoglycemia treatment and midazolam for seizure termination are used when an immediate pharmacologic intervention is required.^23^, ^26^ Successful administration of IN products in treating a variety of conditions provides promising support for IN epinephrine in the treatment of anaphylaxis.

Despite the use of IN administration of drugs for various indications and the known advantages of IN administration, studies on the potential use of IN epinephrine in the treatment of anaphylaxis are lacking. To our knowledge, only one preliminary clinical study has been conducted, which investigated epinephrine pharmacokinetic (PK) after IN versus IM administration in five participants.^27^ Likewise, only two preclinical studies on IN epinephrine have been published, which were conducted in dogs with compromised cardiovascular activity during ventricular fibrillation.^28^, ^29^ We have conducted the current study in dogs to expand upon the literature knowledge base on IN epinephrine, and to provide the platform for future clinical studies.

The current study evaluated the PK profile of varying doses of epinephrine via IN administration in dogs. The study aimed to compare PK parameters, heart rate effects, and safety of IN epinephrine via nasal drops versus IM epinephrine via autoinjector.

## 2 MATERIALS AND METHODS

### 2.1 Animals

All animal experiments were approved by the Institutional Animal Care and Use Committee of MRIGlobal prior to dog procurement from a US Department of Agriculture (USDA)‐certified vendor. Dogs aged 10 to 11 months (7.6 to 10.5 kg) from Covance Research Products were used in the study. Dogs were individually housed indoors in primary enclosures (cage banks, Shor‐line) that provided floor space either meeting or exceeding specifications of the USDA Animal Welfare Act and as described in the Guide for the Care and Use of Laboratory Animals.^30^, ^31^ Dogs were housed under controlled environmental conditions with a standard 12‐hour light/dark cycle, with free access to food and water, and exercised on a regular weekly basis. Prior to epinephrine administration, dogs were shaved on the left and right chest for electrode placement and on the right thigh for IM injections.

### 2.2 Experimental Design

The primary aim of these studies was to determine if escalating doses of IN epinephrine in dogs would result in a corresponding increase in plasma epinephrine concentration as well as physiologic increase in heart rate. Single doses of IN epinephrine were administered to the nostril of each dog at doses of 2, 3, 4, 5, 10, and 20 mg, with a 24‐hour washout period, if necessary. Dogs were administered a single IM epinephrine dose of either 0.15 or 0.3 mg to the bicep femoris muscle with autoinjectors (EpiPen^®^, Mylan, Canonsburg, PA). Three plasma samples were collected for PK analysis at the pre‐dose phase (60, 12, and 1 minute prior to time zero) and after epinephrine administration at 1, 5, 10, 15, 20, 30, 60, and 90 minutes. The PK parameters measured for absorption and exposure included the maximum plasma concentration (C_max_), time to reach maximum plasma concentration (T_max_), and area under the plasma concentration‐time curve from 0 to 90 minutes (AUC_0–90_). Electrocardiogram (ECG) telemetry measurements for corresponding pharmacodynamic analysis were obtained at the same three pre‐dose time points and after epinephrine administration at 1, 5, 10, 15, 20, 30, 60, and 90 minutes. Clinical observations were performed at 60 minutes pre‐ and post‐administration of IN and IM epinephrine.

### 2.3 Formulation Components

The epinephrine used for IN administration was purchased from MilliporeSigma, stored at 5 ± 3°C, and protected from light. The vehicle for IN epinephrine was formulated at MRIGlobal and was based on the injectable product with appropriate modifications suitable for IN administration. In addition to water, sodium metabisulfite (SMBS) and sodium chloride, the formulation included a viscosity modifier, preservative, and buffer. The final formulation had a pH of 5.0 ± 0.5. Autoinjectors were procured from local pharmacies and stored at room temperature (20‐25°C) and protected from light.

### 2.4 Dosing

For IN administration, conscious dogs (no anesthesia/sedation) were restrained by a technician, while a second technician delivered 100 µL of epinephrine in a 200‐µL capacity cannula (pipette tip) that was attached to a 100‐µL calibrated pipette. No dead space was present in the cannula following dose delivery. The entire dose was delivered at a depth of three‐quarters of an inch into the right nostril. For IM epinephrine administration, the autoinjector dosing procedure provided in the manufacturer's instructions was utilized.

### 2.5 Sample collection

Up to 2 mL of whole blood was collected (Vacuette^®^ tube 4 mL K2EDTA [Greiner Bio‐One]) via venous puncture from jugular or cephalic vein. Blood serum and plasma samples were separated from whole blood by centrifugation, kept on ice, and protected from light when possible during the collection, aliquoting, and transfer processes. The PK plasma samples were vortex‐mixed for approximately 1 minute, followed by centrifugation and aliquoting (typically 3 × 100 μL) into amber microcentrifuge tubes containing SMBS (5 μL). Following mixing, samples were transferred to the MRIGlobal Bioanalytical Group for analysis (one aliquot per PK sample).

### 2.6 Bioanalysis of plasma samples

Plasma samples were analyzed for epinephrine concentrations using a calibrator range (lower limit of quantitation to upper limit of quantitation) of either 0.4 to 10 ng/mL or 1 to 32 ng/mL. Samples used for quality control (QC) were 0.4, 1, 3, and 5 ng/mL, and 1, 4, 12, and 24 ng/mL, respectively. Control plasma was heat treated (55°C for ~ 8 days) and stabilized with SMBS (~ 317 mg/mL) (plasma/SMBS = 98:2, volume/volume) before use. Calibrators, QCs, blanks, and incurred samples were prepared by solid‐phase extraction using Biotage Evolute^®^ Express WCX (10 mg) 96‐well plates (Uppsala, Sweden) and epinephrine‐d6 as a true internal standard. The obtained extracts were analyzed by liquid chromatography tandem‐mass spectrometry (LC‐MS/MS) using a C18‐PFP column. Analysis with LC‐MS/MS was performed in positive electrospray ionization mode using multiple reaction monitoring ionization.

### 2.7 Statistical analysis

Epinephrine plasma concentrations were adjusted to account for the plasma epinephrine baseline concentration by subtracting the average concentration of three pre‐dose samples (1, 12, and 60 minutes prior to time 0) from the post‐dose values for each dog. In addition, epinephrine concentrations were considered as outliers and removed from analysis if they exceeded two times the standard deviation from the mean of baseline‐subtracted post‐dose epinephrine plasma concentrations of each dog over the course of blood sampling (1 to 90 minutes post‐dose).

For the IN epinephrine groups, the elimination phase could not be defined; thus, the trapezoidal rule (GraphPad Prism 7.0c) was used to calculate AUC_0–90_. For IM epinephrine groups, the elimination phase could be defined; thus, AUC_0–90_ was calculated using both compartmental and noncompartmental analyses. A comparison of the PK parameters was accomplished with the use of Student's *t *test group comparisons with the level of probability set at < 0.05. All values are expressed as the mean ± standard error.

### 2.8 ECG telemetry

Dogs were fitted with Lomir Biomedical Inc^®^ (Malone, NY) telemetric collection jackets equipped with emkaPACK4G devices (Sterling, VA) to measure heart rate or ECG activity. ECGs were recorded 60 minutes prior to dosing and continuously through 90 minutes post‐dose. Heart rates were reported from IOX (emka Technologies) to correspond with PK time points. Clinical observations were performed 60 minutes pre‐ and post‐dose administration. All values are expressed as the mean ± standard error.

### 2.9 Safety

All dogs were monitored regularly by the veterinarian on‐site for adverse events, or occurrences including illness or reaction with or without the presence of study drug, as per the USDA Veterinary Dictionary for Drug Related Affairs (VeDDRA) current guidance.^32^ Specifically, adverse events were recorded in log books at approximately 1 hour before and after epinephrine administration, and throughout the duration of the study.

## 3 RESULTS

### 3.1 PK of IN versus IM epinephrine

Twenty dogs (10 male and 10 female) were included in the study, with six in each treatment group, aside from the 20‐mg IN epinephrine group (n = 5). The C_max_, T_max_, and AUC_0–90_ values for all treatment groups are presented in Table 1. Greater variability in PK parameters was demonstrated after IN epinephrine versus IM epinephrine, particularly at the lower doses administered. The C_max_, T_max_, and AUC_0–90_ values were inconsistent after 2‐, 3‐, 4‐, and 5‐mg IN epinephrine, and then trended toward dose‐proportional increases with 10‐ and 20‐mg IN epinephrine.

**Table 1:** PK parameters following IN or IM administration of epinephrine

PK parameter^a^	IN epinephrine						IM epinephrine	
	2 mg n = 6	3 mg n = 6	4 mg n = 6	5 mg n = 6	10 mg n = 6	20 mg n = 5	0.15 mg n = 6	0.3 mg n = 6
C_max_ (ng/mL)	2.79 ± 0.96	2.37 ± 1.26	3.75 ± 1.71	3.43 ± 0.65	8.28 ± 1.97	23.28 ± 8.71	1.25 ± 0.19	2.81 ± 0.97
T_max_ (minutes)	37.00 ± 15.48	20.17 ± 14.10	48.50 ± 13.15	41.67 ± 15.95	15.00 ± 3.42	15.20 ± 11.23	21.83 ± 8.74	31.67 ± 9.37
AUC_0–90_ (ng*minutes/mL)	95.59 ± 41.39	91.23 ± 41.35	192.49 ± 99.49	153.19 ± 20.13	207.56 ± 55.72	660.61 ± 323.75	58.93 ± 6.64	118.43 ± 19.40

Abbreviations: AUC_0–90_, area under the plasma concentration‐time curve from 0 to 90 minutes; C_max_, maximum plasma concentration; IM, intramuscular; IN, intranasal; PK, pharmacokinetic; T_max_, time to reach maximum plasma concentration.

^a^ Results are reported as mean ± standard error.

The C_max_ values for 2‐mg IN and 0.15‐mg IM epinephrine were not significantly different (n = 6; 2.79 ± 0.96 ng/mL vs 1.25 ± 0.19 ng/mL, *P* = .15). Likewise, there was not a significant difference in T_max_ with 2‐mg IN vs 0.15‐mg IM epinephrine (n = 6; 37.00 ± 15.48 minutes vs 21.83 ± 8.74 minutes, *P* = .41). The AUC_0–90_ values for 2‐mg IN and 0.15‐mg IM epinephrine were also not significantly different (n = 6; 95.59 ng*minutes/mL ± 41.39 vs 58.93 ± 6.64 ng*minutes/mL, *P* = .40).

There was not a significant difference in C_max_ between 4‐mg IN and 0.3‐mg IM epinephrine (n = 6, 3.75 ± 1.71 ng/mL vs 2.81 ± 0.97 ng/mL, *P* = .64) or 5‐mg IN and 0.3‐mg IM epinephrine (n = 6, 3.43 ± 0.65 ng/mL vs 2.81 ± 0.97 ng/mL, *P* = .61). The T_max_ values for 4‐mg or 5‐mg IN and 0.3‐mg IM epinephrine were also not significantly different (n = 6, 48.50 ± 13.15 minutes vs 31.67 ± 9.37 minutes, *P* = .32; n = 6, 41.67 ± 15.95 minutes vs 31.67 ± 9.37 minutes, *P* = .60). Lastly, there were no significant differences in AUC_0–90_ between 4‐ or 5‐mg IN and 0.3‐mg IM epinephrine (n = 6, 192.49 ± 99.49 ng*minutes/mL vs 118.43 ± 19.40 ng*minutes/mL, *P* = .48; n = 6, 153.19 ± 20.13 ng*minutes/mL vs 118.43 ± 19.40 ng*minutes/mL *P* = .24).

**Figure 1:**
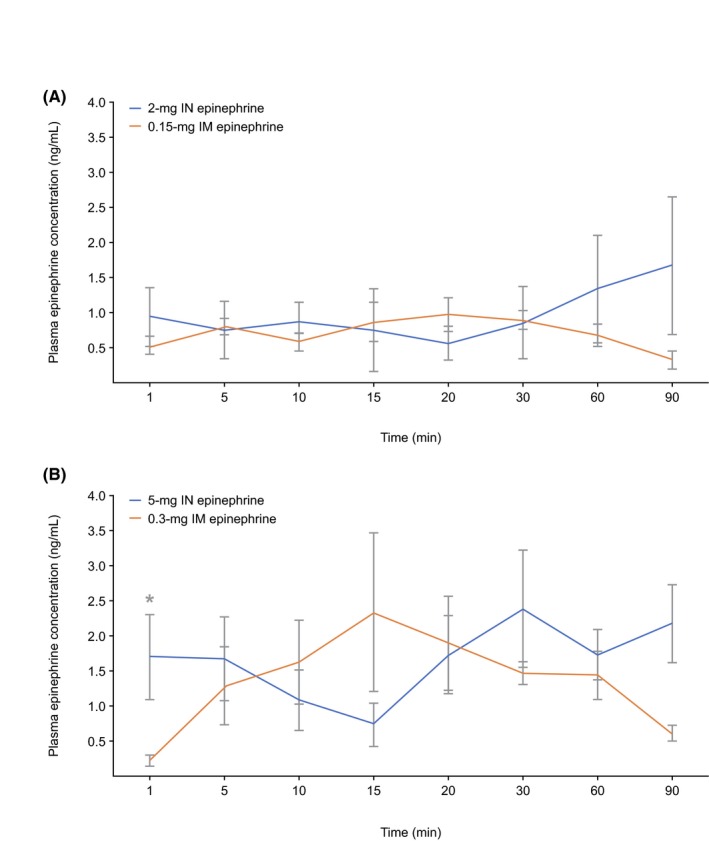
Average epinephrine plasma concentration over time following 2 mg‐IN and 0.15‐mg IM epinephrine (A), and 5‐mg IN and 0.3‐mg IM epinephrine (B). Epinephrine administration (epinephrine plasma concentrations were adjusted to account for the plasma epinephrine baseline by subtracting the average concentration of three pre‐dose samples (60, 12, and 1 minute prior to time 0) from the post‐dose values for each dog.) ^*^ *P* < .05. IM, intramuscular; IN, intranasal

Within 1‐minute post‐dose, the average plasma epinephrine concentration was numerically greater with 2‐mg IN epinephrine than with 0.15‐mg IM epinephrine (0.94 ± 0.48 ng/mL vs 0.53 ± 0.13 ng/mL, *P* = .39) (Figure 1A). After 2‐mg IN epinephrine dosing, plasma epinephrine concentration slightly decreased at 20 minutes, then steadily increased up to 90 minutes. After 0.15‐mg IM epinephrine dosing, plasma epinephrine peaked at 20 minutes, then gradually declined over 90 minutes.

At 1 minute, 5‐mg IN epinephrine produced a 7‐fold greater epinephrine concentration than 0.3‐mg IM epinephrine (1.68 ± 0.65 ng/mL vs 0.21 ± 0.08 ng/mL, *P* = .03; Figure 1B), with a concentration of 41% of the C_max_ versus 7% after 0.3‐mg IM epinephrine. After 5‐mg IN epinephrine, the plasma epinephrine concentration decreased 5 to 15 minutes after administration, then began increasing at 20 minutes, peaked at 30 minutes, then remained stable for up to 90 minutes. After 0.3‐mg IM epinephrine, plasma epinephrine concentration peaked at 15 minutes, and then gradually declined over 90 minutes.

Administration of 10‐mg IN epinephrine resulted in a plasma concentration of 4.09 ± 1.56 ng/mL after 1 minute, with an increase to 4.63 ± 3.11 ng/mL at 5 minutes. Administration of 20‐mg IN epinephrine resulted in plasma epinephrine concentrations of 6.08 ± 3.21 ng/mL and 16.34 ± 9.58 ng/mL at 1 and 5 minutes post‐dose, respectively. There were no statistical comparisons performed for the 10‐ and 20‐mg IN epinephrine doses.

### 3.2 Cardiovascular effects

**Figure 2:**
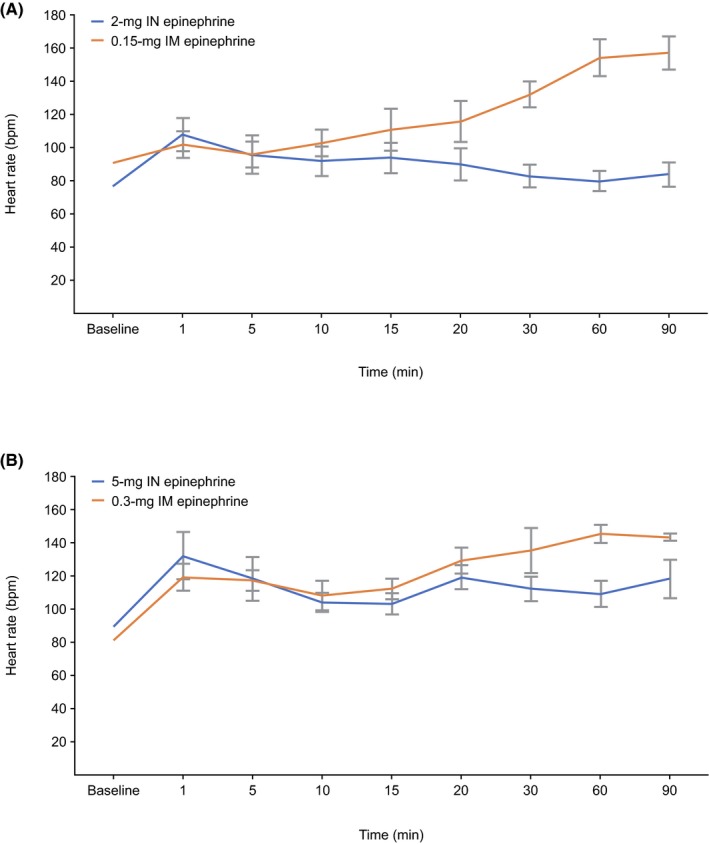
Average heart rate over time following IN or IM epinephrine administration. bpm, beats per minute; IM, intramuscular; IN, intranasal

Within 1 minute of 2‐mg IN epinephrine, heart rate increased to 108 ± 10.08 beats per minute (bpm) (40% over baseline) (Figure 2A). This reduced to a sustained level of 25% elevation from baseline for the next 20 minutes and decreased to less than 10% elevation at 30 minutes. Within 1 minute following 0.15‐mg IM epinephrine administration, heart rate increased to 102 ± 8.22 (12% above baseline). At 15 minutes following 0.15‐mg IM epinephrine, heart rate was elevated to 111 ± 12.88 (22% above baseline) and continued to increase to 157 ± 10.06 (73%) at 90 minutes.

Within the first minute following 5‐mg IN epinephrine, heart rate increased to 132 ± 14.34 bpm (48% over baseline) (Figure 2B). However, the increase was of relatively short duration, with heart rates declining to 36% over baseline within 5 minutes. The magnitude of the heart rate change observed at 5 minutes remained relatively constant over the next 90 minutes. Within the first minute following 0.3‐mg IM epinephrine administration, heart rate increased to 47% over baseline. The level of heart rate increase remained relatively constant for the next 15 minutes and began to steadily increase by 59% over baseline at 20 minutes with a further increase to 79% over baseline at 60 and 90 minutes.

### 3.3 Safety

IN epinephrine (2 to 20 mg) did not result in the appearance of either immediate or sustained adverse events in dogs that were followed for 2 weeks following the last drug administration. The most common adverse events with IN epinephrine were hypersalivation and emesis (Table 2). The most common adverse event with IM epinephrine was limping. Dogs exhibited signs of stress following IN and IM drug administration; however, all recovered within several hours, and no sustained effects were noted over the following 2 weeks.

**Table 2:** Adverse events observed for up to 2 weeks after IN or IM epinephrine administration

Observation, n	IN epinephrine						IM epinephrine	
	2 mg n = 6	3 mg n = 6	4 mg n = 6	5 mg n = 6	10 mg n = 6	20 mg n = 5	0.15 mg n = 6	0.3 mg n = 6
Limping	0	0	0	0	0	0	0	3
Hypersalivation	1	2	0	1	3	4	0	1
Red nasal discharge	0	1	0	1	0	0	0	0
Salivary staining (pink)	0	1	0	0	0	0	0	0
Emesis	0	0	0	1	0	2	0	0

Abbreviations: IM, intramuscular; IN, intranasal.

## 4 DISCUSSION

In this study of the PKs of IN epinephrine dosing in dogs, epinephrine was rapidly absorbed at 1 minute after IN as compared to IM administration. Overall, PK parameters were similar between IN and IM epinephrine, with a trend for dose‐dependent increases in C_max_ and AUC_0‐90_ after IN and IM epinephrine, with some variability in dose‐response after IN epinephrine. The C_max_ and AUC_0‐90_ values changed inconsistently after 2‐, 3‐, and 4‐mg IN epinephrine, and the increases after 10‐ and 20‐mg IN epinephrine were greater than dose‐proportional effects. Increased heart rate was sustained after IM epinephrine over the course of 90 minutes, as compared to IN epinephrine, which showed reductions of increased heart rate over time. No differences in safety measures were indicated between IN and IM epinephrine at the time of administration, and for up to 2 weeks of follow‐up.

Plasma epinephrine concentration 1 minute after dosing was significantly greater with 5‐mg IN vs 0.3‐mg IM epinephrine, followed by a brief reduction in plasma epinephrine at 10 to 15 minutes and a subsequent sustained increase in epinephrine concentration for up to 90 minutes (2.15 ng/mL). The classical bell‐shaped plasma concentration‐time curve after 0.3‐mg IM epinephrine showed an initial increase in epinephrine concentration during absorption, followed by a plateau phase, and then a decrease in epinephrine concentration, reflecting distribution and elimination at 90 minutes (0.6 ng/mL).^33^

The heart rate increase after IN epinephrine decreased 15 to 20 minutes after 2‐ and 5‐mg IN epinephrine, while heart rate remained elevated for up to 90 minutes after 0.15‐ and 0.3‐mg IM epinephrine. In addition, the dose‐response relationships between epinephrine administration and increased heart rate differed between IN and IM epinephrine. The significantly increased epinephrine plasma concentration observed 1 minute after 5‐mg IN epinephrine corresponded with a heart rate increase of 48% from baseline. Increased heart rate following 5‐mg IN epinephrine decreased 5 minutes after IN administration, and remained decreased over the 90‐minute period. After 0.15‐ and 0.3‐mg IM epinephrine, heart rate continued to increase over time, with the greatest increases at 30 to 90 minutes after administration. Increased heart rate after IM epinephrine corresponded to decreasing epinephrine concentrations at 30 to 90 minutes, suggesting a lack of correlation between plasma epinephrine concentration and heart rate. The established variability of epinephrine uptake may explain the inconsistent dose‐response relationship between epinephrine and heart rate effects.^14^

In addition to epinephrine uptake variability, receptor subtype‐specific effects may be responsible for the inconsistent plasma concentration‐time curve and heart rate effects after IN epinephrine.^14^, ^34^ For example, the brief reductions in plasma epinephrine 5 to 20 minutes after 2‐mg IN epinephrine dosing and 5 to 15 minutes after 5‐mg IN epinephrine dosing were likely due to the alpha‐adrenergic receptor‐mediated vasoconstrictive properties of epinephrine potentially evident only after IN administration.^34^ The sustained increase in plasma epinephrine concentration for 90 minutes after 5‐mg IN epinephrine is not likely from the result of accumulation over time because the half‐life of epinephrine is 2 to 3 minutes.^35^ Differences in heart rate activity may also be explained by variability in epinephrine absorption and differential activation of beta‐adrenergic receptors that mediate tachycardia.^35^ The inconsistent pattern of plasma epinephrine concentrations and heart rate effects may be supported by the known variability in epinephrine absorption across a range of doses.^14^, ^27^ Further studies are required to understand the irregular plasma concentration‐time curve and heart rate dose‐response relationship after IN epinephrine.

Though there are many known advantages of IN administration, the literature regarding the use of IN epinephrine to treat anaphylaxis is limited. There are only a few studies that have investigated IN epinephrine.^27^, ^28^, ^29^ Two preclinical studies showed that IN epinephrine rapidly increased plasma epinephrine concentrations and improved coronary blood flow during cardiopulmonary resuscitation in dogs.^28^, ^29^ These studies required the use of higher doses of epinephrine (7.5 to 14 mg) due to the compromised cardiovascular system of the dogs during ventricular fibrillation. In these studies, an alpha‐adrenergic blocker, phentolamine, was administered prior to IN epinephrine to improve epinephrine absorption.^28^, ^29^

We have also used dogs to study epinephrine PK and pharmacodynamics after IN versus IM administration. Dogs are a favorable species for clinical translation due to the similarities in the nasal environment between dogs and humans, such as similar nasal volume, dimension, surface area, and clearance.^36^, ^37^ We have demonstrated that it is not necessary to administer an alpha‐adrenergic blocker in conjunction with IN epinephrine to achieve epinephrine absorption via the IN route. In addition, we have used a wide range of IN epinephrine doses that were expected to equate epinephrine absorption as compared to the FDA‐approved IM doses contained within the autoinjectors. Because the bioavailability of polar substances like epinephrine through the nasal mucosa is relatively low,^18^, ^38^ we chose to include a range of epinephrine doses for comparison to 0.15‐ and 0.3‐mg IM epinephrine. Unlike other studies, the formulation for IN epinephrine used in the current study was specifically designed to include an absorption enhancer in place of distilled water, along with a viscosity agent and buffer to optimize epinephrine stability and delivery.^27^

A preliminary clinical study evaluated comparative bioavailability of IN versus IM epinephrine in five adult participants. This pilot clinical study demonstrated bioequivalence between 5‐mg IN epinephrine and 0.3‐mg IM epinephrine, with comparable C_max_, AUC_0‐120_, and T_max_ after IN and IM epinephrine.^27^ Further clinical studies with more participants are required to confirm these findings of bioequivalence between IN and IM epinephrine. Future clinical studies in humans will aim to investigate the administration of the IN epinephrine formulation used in the current study and to validate the hypothesis that IN epinephrine leads to epinephrine absorption with bioequivalence to IM epinephrine. In addition, we have ongoing preclinical and clinical studies evaluating IN epinephrine in the context of severe allergy or anaphylaxis.

IN epinephrine administration offers several potential advantages over IM administration in the treatment of anaphylaxis. First, 1 minute after dosing, IN epinephrine showed a significantly greater plasma epinephrine concentration versus IM epinephrine. This 1‐minute difference in epinephrine concentration may be a critical factor in the potential treatment of a patient with anaphylaxis because mismanagement of anaphylaxis with delayed recognition of allergic symptoms or delayed treatment with epinephrine can be fatal.^3^, ^39^, ^40^, ^41^, ^42^ Indeed, there is a negative correlation between the time to epinephrine treatment and a patient's rate of survival.^17^ Thus, immediate treatment and epinephrine absorption is crucial in the treatment of anaphylaxis. The second advantage of IN administration of epinephrine is that this route provides more sustained and consistent plasma epinephrine concentrations over time. Third, the trend toward an increased dose effect presents an advantage of heightened dose administration with IN epinephrine. Fourth, IN epinephrine resulted in decreased heart rate effects compared to IM epinephrine, which may translate to a reduced risk of tachycardia during the treatment of anaphylaxis.

Other advantages of IN epinephrine include its portability and convenient administration method, which may increase the potential for carrying the device more regularly and decrease user anxiety, thereby decreasing the risk of delayed treatment.^10^, ^43^ IN administration avoids accidental finger sticks or skin lacerations that may be experienced during IM administration.^7^, ^9^ Lastly, IN epinephrine may be a cost‐effective alternative treatment strategy. Costs for IN naloxone sprays have remained stable over the course of 10 years (2006 to 2017) with increasing demand, while the costs of all other naloxone formulations, including IM formulations, have increased.^44^ A retrospective US claims study found that a nasal spray for allergic rhinitis had better economic outcomes for patients than combinations of agents, and appeared to keep asthma‐related health‐care costs down.^45^

In the current preclinical study, IN epinephrine produced rapid epinephrine absorption and similar PK parameters overall as compared to IM epinephrine. Additionally, IN epinephrine resulted in blunted heart rate effects as compared to IM epinephrine, and was well‐tolerated. Clinical studies will further investigate IN epinephrine PK and pharmacodynamic effects. IN epinephrine may be a potential alternative therapeutic approach in the treatment of severe allergy and anaphylaxis.

## 5 ETHICS STATEMENT

All animal experiments were approved by the Institutional Animal Care and Use Committee of MRIGlobal (Kansas City, MO) prior to dog procurement from a US Department of Agriculture (USDA)‐certified vendor.

## DISCLOSURES

The authors have no conflict of interest to declare.

## AUTHOR CONTRIBUTIONS

*Participated in research design:* Dretchen, Z Mesa, Robben, Slade, Hill, Croutch, Kappeler. *Conducted experiments:* Dretchen, Z Mesa, Slade, Hill, Croutch, Kappeler. *Performed data analysis:* Dretchen, Z Mesa, Slade, Hill, Croutch, Kappeler. *Wrote or contributed to the writing of the manuscript:* Dretchen, Z Mesa, Robben, Slade, Hill, Croutch, Kappeler, M Mesa.
